# Correction: Life-threatening fetal goiter: diagnosis and outcomes in four consecutive cases with critical appraisal—why this diagnosis is not obviously trivial

**DOI:** 10.1007/s00404-026-08562-5

**Published:** 2026-11-02

**Authors:** Jann Lennard Scharf, Michael Gembicki, Amrei Welp, Guido Stichtenoth, Verena Sailer, Jan Weichert

**Affiliations:** 1 Department of Gynecology and Obstetrics, Division of Prenatal Medicine, University of Luebeck, University Hospital Schleswig-Holstein, Campus Luebeck, Ratzeburger Allee 160, 23538 Luebeck, Germany; 2 Department of Pediatrics, University of Luebeck, University Hospital Schleswig-Holstein, Campus Luebeck, Ratzeburger Allee 160, 23538 Luebeck, Germany; 3 Institute of Pathology, University of Luebeck, University Hospital Schleswig-Holstein, Campus Luebeck, Ratzeburger Allee 160, 23538 Luebeck, Germany; 4 Elbe Center for Prenatal Medicine and Human Genetics, Willy-Brandt-Str. 1, 20457 Hamburg, Germany

**Correction: Archives of Gynecology and Obstetrics (2026) 313:248** **https://doi.org/10.1007/s00404-026-08543-8**

In this article, Table 1 and Table 2 appeared incorrectly and have now been corrected in the original publication. For completeness and transparency, the correct and old incorrect versions are displayed below.

Incorrect Table 1

**Table 1:** Key characteristics of gravidae and fetuses/neonates in cases no. 1 to 4

Characteristics	Cases			
	no. 1	no. 2	no. 3	no. 4
Characteristics of gravidae Age (years) 2 Pre-pregnancy BMI (kg/m ) Maternal thyroid status GA at diagnosis of fetal goiter (wks) GA at delivery (wks) Delivery mode	38	31	22	28
	20.30	19.10	31.25	26.99
	Graves’ disease Euthyroid	Mild central hypothyroidism	Latent hypothyroidism	Euthyroid
	35+5	25+1	20+5	27+5
	38+0	34+3	31+1	34+3
	Cesarean section	Cesarean section	Cesarean section	Cesarean section
Characteristics of fetuses/neonates				
Birth weight (g)	3,170	2,230	1,745	1,950
Umbilical artery cord pH	7.24	7.12	7.15	-
APGAR (1/5/10 min)	4/6/5	7/8/8	1/1/1	0/0/0
Outcome	Postpartum death	Live birth	Postpartum death	Fetal demise
Gender	Male	Female	Male	Male
Autopsy	Yes	Not required	Yes	No

*BMI* body mass index; *GA* gestational age; *wks*:weeks of gestation

Correct Table 1

**Table 1:** Key characteristics of gravidae and fetuses/neonates in cases no. 1 to 4

Characteristics	Cases			
	No. 1	No. 2	No. 3	No. 4
Characteristics of gravidae				
Age (years)	38	31	22	28
Pre-pregnancy BMI (kg/m^2^)	20.30	19.10	31.25	26.99
Maternal thyroid status	Graves’ disease Euthyroid	Mild central hypothyroidism	Latent hypothyroidism	Euthyroid
GA at diagnosis of fetal goiter (weeks)	35 + 5	25 + 1	20 + 5	27 + 5
GA at delivery (weeks)	38 + 0	34 + 3	31 + 1	34 + 3
Delivery mode	Cesarean section	Cesarean section	Cesarean section	Cesarean section
Characteristics of fetuses/neonates				
Birth weight (g)	3,170	2,230	1,745	1,950
Umbilical artery cord pH	7.24	7.12	7.15	–
APGAR (1/5/10 min)	4/6/5	7/8/8	1/1/1	0/0/0
Outcome	Postpartum death	Live birth	Postpartum death	Fetal demise
Gender	Male	Male	Female	Male
Autopsy	Yes	Not required	Yes	No

*BMI* body mass index; *GA* gestational age; *wks*:weeks of gestation

Incorrect Table 2

**Table 2:** Timeline of intrauterine management of fetal goiter in the four cases

Items of treatment	Case No. 1			Case no. 2										Case no. 3									Case no. 4									
course	GA in wks		At birth	Follow-up with GA in wks									At birth	Follow-up with GA in wks								At birth	Follow-up with GA in wks									At birth
	35+5	38+0		25+1	26+5	28+0	28+5	30+0	31+1	32+5	34+1	34+3		20+5	21+5	24+5	25+1	26+1	28+1	29+1	31+1		27+5	28+5	29+1	30+2	31+6	32+6	34+3			
**Initial diagnosis**	x			x										x									x									
**Intrauterine procedure**																																
Cordocentesis							x								x					x							x					
Amniocentesis							x		x		x				x		x			x					x	x	x	x				
Administration of LT4 (µg/injection)							200		250		250				250		250			300					250	250	300	350				
**Monitoring** 3 Goiter volume* (cm ) by ultrasound Goiter size by fetal MRI Left lobe (cm) Right lobe (cm)	22.36			12.74	x1.1 x1.0	15.34	16.23	9.42	10.11	9.56	8.22		6.48	4.38	4.76	7.88		5.37	4.73				17.24	24.24	19.55	15.19	28.93	23.67				
**Fetal thyroid status** TSH (mIU/L)							>100		>100				31.2							>100			>100									
Fetal bloodfT3 (pmol/L)							1.49		1.9				3.16							1.5			4.86									
fT4 (pmol/L)							5.1		6.6				36.7							4.7			9.9									
TSH (mIU/L)							11.2								8.27		5.64															
Amniotic fluidfT3 (pmol/L)							1.19								1.69		2.01															
fT4 (pmol/L)							5.6								3.8		12.4															
**Maternal thyroid status**																																
TSH (mIU/L)	5.5					0.595				0.431				3.63									1.63		1.88	3.16						
fT3 (pmol/L)	5.11									3.46				5.04											4.35							
fT4 (pmol/L)	5.9									13.3				10.5											13.4							
Tg (µg/L)	4.03					55.60																										
TgAb (kIU/L)						<10.00																			17.4							
TPOAb (kIU/L)	<10.00					<9.00																			15.2							
TRAb (IU/L)	1.7													<0.8											1.24							
**Karyotype**				46, XY										46, XX									46, XY									
**Delivery**	x			x										x									x									
**3** **Goiter volume (cm ) by autopsy**			33.65																			23.76										

*GA* gestational age; *WG* weeks of gestation; *MRI* magnetic resonance imaging; *TSH* thyroid-stimulating hormone; *fT3* free triiodothyronine; *fT4* free thyroxine; *Tg* thyroglobulin; *TgAb* thyroglobulin antibody; *TPOAb*: thyroid peroxidase antibody; *TRAb* thyrotropin receptor antibody. *GA* gestational age; *wks*: weeks of gestation

^*^Volume calculation was performed idealized measuring both thyroid lobes while ignoring the isthmus. Maternal thyroid status in early gestation in case no. 1: TSH < 0.004 mIU/L, fT3 16.7 pmol/L, fT4 31.2 pmol/L, Tg 0.68 µg/L, TPOAb < 10.00 kIU/L, TRAb 48.5 IU/L

Correct Table 2

**Table 2:** Timeline of intrauterine management of fetal goiter in the four cases

Items of treatment course		Case no. 1			Case no. 2										Case no. 3									Case no. 4							
		GA in weeks		At birth	Follow-up with GA in weeks									At birth	Follow-up with GA in weeks								At birth	Follow-up with GA in weeks							At birth
		35 + 5	38 + 0		25 + 1	26 + 5	28 + 0	28 + 5	30 + 0	31 + 1	32 + 5	34 + 1	34 + 3		20 + 5	21 + 5	24 + 5	25 + 1	26 + 1	28 + 1	29 + 1	31 + 1		27 + 5	28 + 5	29 + 1	30 + 2	31 + 6	32 + 6	34 + 3	
Initial diagnosis		x			x										x									x							
Intrauterine procedure																															
Cordocentesis								x								x					x							x			
Amniocentesis								x		x		x				x		x			x					x	x	x	x		
Administration of LT4 (µg/injection)								200		250		250				250		250			300					250	250	300	350		
Monitoring																															
Goiter volume^a^ (cm^3^) by ultrasound		22.36			12.74		15.34	16.23	9.42	10.11	9.56	8.22		6.48	4.38	4.76	7.88		5.37	4.73				17.24	24.24	19.55	15.19	28.93	23.67		
Goiter size by fetal MRI																															
Left lobe (cm)						1.2 × 1.1																									
Right lobe (cm)						1.3 × 1.0																									
Fetal thyroid status																															
Fetal blood	TSH (mIU/L)							>100		>100				31.2							>100							>100			
	fT3 (pmol/L)							1.49		1.9				3.16							1.5							4.86			
	fT4 (pmol/L)							5.1		6.6				36.7							4.7							9.9			
Amniotic fluid	TSH (mIU/L)							11.2								8.27		5.64													
	fT3 (pmol/L)							1.19								1.69		2.01													
	fT4 (pmol/L)							5.6								3.8		12.4													
Maternal thyroid status																															
TSH (mIU/L)		5.5					0.595				0.431				3.63									1.63		1.88	3.16				
fT3 (pmol/L)		5.11									3.46				5.04											4.35					
fT4 (pmol/L)		5.9									13.3				10.5											13.4					
Tg (µg/L)		4.03					55.60																								
TgAb (kIU/L)							<10.00																			17.4					
TPOAb (kIU/L)		<10.00					<9.00																			15.2					
TRAb (IU/L)		1.7													<0.8											1.24					
Karyotype								46, XY								46, XX										46, XY					
Delivery			x										x									x								x	
Goiter volume (cm^3^) by autopsy				33.65																			23.76								

*GA* gestational age; *Wks* weeks of gestation; *MRI* magnetic resonance imaging; *TSH* thyroid-stimulating hormone; *fT3* free triiodothyronine; *fT4* free thyroxine; *Tg* thyroglobulin; *TgAb* thyroglobulin antibody; *TPOAb*: thyroid peroxidase antibody; *TRAb* thyrotropin receptor antibody

^a^Volume calculation was performed idealized measuring both thyroid lobes while ignoring the isthmus. Maternal thyroid status in early gestation in case no. 1: TSH < 0.004 mIU/L, fT3 16.7 pmol/L, fT4 31.2 pmol/L, Tg 0.68 µg/L, TPOAb < 10.00 kIU/L, TRAb 48.5 IU/L

The original article has been corrected.

